# In-situ Measurement of Self-Atom Diffusion in Solids Using Amorphous Germanium as a Model System

**DOI:** 10.1038/s41598-018-35915-1

**Published:** 2018-12-04

**Authors:** Erwin Hüger, Florian Strauß, Jochen Stahn, Joachim Deubener, Michael Bruns, Harald Schmidt

**Affiliations:** 1 AG Mikrokinetik, Institut für Metallurgie, TU Clausthal, Clausthal-Zellerfeld, Germany; 2 Clausthaler Zentrum für Materialtechnik, Technische Universität Clausthal, Clausthal-Zellerfeld, Germany; 3 Laboratory for Neutron Scattering and Imaging, Paul Scherrer Institut, Villigen, Switzerland; 4 Institut für Nichtmetallische Werkstoffe, TU Clausthal, Clausthal-Zellerfeld, Germany; 5 Institute for Applied Materials (IAM) and Karlsruhe Nano Micro Facility (KNMF), Karlsruher Institut für Technologie (KIT), Eggenstein-Leopoldshafen, Germany

## Abstract

We present *in-situ* self-diffusion experiments in solids, which were carried out by Focussing Neutron Reflectometry on isotope multilayers. This new approach offers the following advantages in comparison to classical *ex-situ* measurements: (1) Identification and continuous measurement of a time dependence of diffusivities, (2) significant reduction of error limits of diffusivities, and (3) substantial reduction of the necessary experimental time. In the framework of a case study, yet unknown self-diffusivities in amorphous germanium are measured at various temperatures quasi-continuously, each during isothermal annealing. A significant decrease of diffusivities as a function of annealing time by one order of magnitude is detected that is attributed to structural relaxation accompanied by defect annihilation. In metastable equilibrium the diffusivities follow the Arrhenius law between 375 and 412 °C with an activation energy of Q = (2.11 ± 0.12) eV. The diffusivities are five orders of magnitude higher than in germanium single crystals at 400 °C, mainly due to the lower activation energy.

## Introduction

Self-diffusion can be defined as the thermally activated random migration of atoms in solids. It is a central matter transport process and its understanding is important for the production, the temperature stability, and the functional and structural properties of materials^1^. Self-diffusion may control relevant microstructural re-arrangements like precipitation, crystallization, layer growth during oxidation, plastic deformation^2^, as well as ion-conductivity in electrolytes and electrodes and thus influences physical properties for applications^3^. Concerning these topics, actual research is necessary to prove such correlations. For example, as recently demonstrated the activation enthalpy of self-diffusion in amorphous Si equals the activation enthalpy of solid phase epitaxial recrystallization, pointing to a common mechanism^4^. One of the most important analytical methods for the experimental determination of self-diffusivities and activation energies is based on the use of radioactive or stable tracers (tracer method)^1^. A classical tracer diffusion experiment is done *ex-situ*. First, a small amount of radioactive or rare stable isotope of an element is deposited on the surface of the sample under investigation, e.g. ^73^Ge on ^nat^Ge (stable) or ^71^Ge on ^nat^Ge (radioactive). After annealing at elevated temperature in order to activate diffusion the tracer is redistributed according to Fick’s second law^1^. After quenching the sample to room temperature the atoms become immobile and the redistributed tracer profile is frozen. The isotope profile can now be analyzed in more or less time consuming depth profile analyses with various methods like Secondary Ion Mass Spectrometry (SIMS)^5^, Nuclear Reaction Analysis^6^ or sputter depth profiling of radiotracers^7^ lasting at least several hours. Since more than twenty years isotope heterostructures have been used for studying self-diffusion phenomena in great detail^5,8–12^.

During the last years neutron reflectometry (NR) was established as an analytical method in this field of research to measure self-diffusivities by isotope interdiffusion^13–20^. This method allows determining diffusion lengths on the nanoscale down to below 1 nm. This is especially important for the investigation of amorphous and nanostructured materials due to their intrinsic metastability^21^. The drawback of all tracer methods (radiotracer, SIMS, NR etc.) is that the diffusion process cannot be monitored *in-situ*, directly during annealing. In order to extract diffusivities after tracer deposition, diffusion annealing and tracer redistribution, the sample under investigation has always been cooled down to room temperature in order to freeze diffusion before time-consuming analysis is done. For several advanced metastable materials (amorphous semiconductors, glasses, nano-materials) an *in-situ* recording of diffusivities directly during diffusion and annealing with high temporal resolution in the minutes range is highly interesting. It would allow identifying and quantifying the modification of time-dependent diffusivities due to structural relaxation, crystallization, or grain growth etc. Such effects are often masked by *ex-situ* experiments due to heating and cooling periods and a limited number of data points (diffusivities per time interval). Concerning the NR method, it is not necessary to complexly adjust the sample after each annealing step. This will drastically enhance the exactness of the determination of reflectivities and reduce error limits of diffusivities as well as experimental time. A significantly higher number density of diffusivity values, e.g. as a function of temperature or time, can be reached and activation energies might be determined more precisely.

Due to the actual improvement of the basic NR method in form of “Focussing Reflectometry”^22,23^ it is possible to record a statistically viable reflectivity pattern in about 1 min (instead of hours). This enables a quasi-continuous *in-situ* detection of self-diffusivities directly during annealing. This new approach offers the following advantages: (1) Identification and continuous measurement of time dependent diffusivities, (2) significant reduction of error limits of diffusivities and activation energies, (3) substantial reduction of the necessary experimental time and (4) direct measurement of diffusion during diffusion controlled structural re-arrangements (e.g. precipitation). Here, we present and discuss first experiments on *in-situ* monitoring of self-diffusivities for the model system amorphous germanium in order to illustrate the efficiency of the method.

Amorphous covalently bonded semiconductors are in the focus of experimental and theoretical research for years^24,25^ with applications in the area of microelectronics, optics and energy conversion^26–28^. Classical prototypes are silicon and germanium due to their simple tetrahedral short range structure. The knowledge of diffusion processes in such materials is relatively limited. The high covalent bond energy leads to low self-diffusivities below the crystallization temperature. In addition, the intrinsic metastability has the consequence that annealing (e.g. during preparation or microstructural tailoring) at elevated temperatures may lead to crystallization processes. In this context, an experimental access to such systems is complicate.

Very recently, we were able to successfully measure Si self-diffusivities in sputtered amorphous silicon using *ex-situ* NR^16,29^. Others^4^, determined by SIMS the self-diffusivities in amorphous silicon that was prepared by ion implantation of Si isotope structures on top of a silicon on insulator substrate. For amorphous germanium, self-diffusivities are not known up to now. However, Radek *et al*.^30^ investigated the atomic mixing of an amorphous Ge layer formed by ion self-implantation during solid-phase epitaxial regrowth (SPER) and found an upper limit of 0.5 nm for the displacement length of the Ge atoms.

Amorphous germanium is a non-crystalline modification of classical germanium, which is isostructural to silicon (diamond lattice). The structure can be described as a fourfold coordinated continuous network of covalently bonded Ge atoms without long range order^31,32^. The crystallization temperature is around 450 °C^33–35^ depending on isothermal annealing time. Annealing at elevated temperatures below the crystallization limit may lead to diffusion controlled structural rearrangement processes termed structural relaxation^36–39^. This may alter the point defect density in a direct way and application relevant properties may be modified. Investigations show that structural relaxation can be interpreted also as a defect annihilation processes and a reorganization of bonds^38,40,41^. Such effects will lead to time-dependent diffusivities that have to be identified.

## Methods and Materials

Diffusion experiments were carried out on ^73^Ge/^nat^Ge multilayers. Due to the different coherent neutron scattering lengths of ^73^Ge (5.02 fm) and ^nat^Ge (8.19 fm), a large contrast variation for neutron scattering is given. In the neutron reflectivity pattern artificial Bragg peaks are formed due to the isotope modulation of the multilayer. Self-diffusivities are determined by detecting the decay of the Bragg peaks^15^. The diffusion experiments were done using [^73^Ge (14 nm)/^nat^Ge (14 nm)] × 10 structures, which were produced by ion-beam sputter deposition. The sputter coater (IBC 681, Gatan) is equipped with two Penning sources using Ar as sputter gas. ^nat^Ge and ^73^Ge targets can be installed simultaneously in this setup and used successively without breaking the vacuum. In order to obtain a uniform thickness distribution, the samples were rocked and rotated during the deposition process. The ^nat^Ge layer was sputtered from a disc-shaped polycrystalline germanium target with a diameter of 2 cm (MaTecK, Germany), while ^73^Ge layers were sputtered from a similar but ^73^Ge enriched target (^73^Ge enrichment > 99.3%, MaTecK, Germany). Before each deposition the targets were pre-sputtered to remove possible atmospheric contaminants. The sputtering was done using argon as a sputter gas at a base pressure below 5 × 10^−7^ mbar, an acceleration voltage of 5 kV and a beam current of 180 µA. As substrates (100) oriented, polished, nominally undoped Germanium wafers (CrysTec, Germany) were used, which were cleaned with isopropanol beforehand. The as-deposited multilayers have an overall thickness of 280 nm. Additional annealing was not applied to the deposited amorphous layers in the sputter chamber before removal and handling in air. The temperature of the Ge substrate wafer during deposition was not measured due to experimental difficulties with the rotating sample holder. However, according to literature, the self-heating of the sample during ion-beam sputter experiments is generally low (below 80 °C)^42,43^ due to the low impact energy (tens of eV) of ions which are deposited^44^. The native oxide layer was not removed. Since we used commercial wafers it is expected to be about 2–3 nm as stated in refs^45,46^.

The multilayers were investigated by X-ray photoelectron spectroscopy (XPS) using a K-Alpha spectrometer (ThermoFisher Scientific, East Grinstead, UK) at Karlsruher Institut für Technologie. After removing several tens of nanometers from the sample by Ar ion sputtering, high resolution spectra were recorded. In addition to the main element germanium, a low residual concentration of oxygen of 0.6 at.% and of Fe of 0.5 at.% was detected. The C concentration is below the detection limit of this element of 0.5 at.%. Further, we did a mass scan by SIMS (O_2_^+^ primary beam, 5 keV, Cameca ims 3 f/4 f) for the detection of impurities, using Fig. 3.3A in ref.^47^ for analysis. The C concentration could be assessed to <0.03 at.%. Concerning metals, we found also only Fe with a concentration of 10^−3^ at.% (10 ppm) above the detection limit of the machine of 1 ppm. The underestimation of the Fe concentration by SIMS analysis is attributed to the unknown relative sensitivity factors of amorphous germanium and the not very well documented relative sensitivity factors of crystalline germanium^47^. Since the main topic of this work is to illustrate the advantages of the *in-situ* approach, this impurity level is tolerable at a first step. It might be interesting to compare the results to very high purity amorphous germanium as prepared e.g. by ion self-implantation^30^.

NR was carried out at the reflectometer AMOR located at SINQ (Paul Scherer Institute, Villigen, Switzerland). During NR experiments, a neutron beam strikes the sample surface under a small angle, θ, is reflected at each isotope interface and afterwards detected. Due to the superposition of the individual partial beams a characteristic interference pattern is formed (Bragg peak). The intensity of the reflected beam is measured as a function of the scattering vector normal to the surface q_z_ = 4π sin θ λ^−1^ (λ: neutron wavelength). The *in-situ* NR measurements were done with the Selene focussing optics^22,23^ recently implemented at AMOR. For standard NR methods, either the angle, θ, or the wavelength, λ, is kept fixed and the other quantity is varied. The Selene setup allows a variation of both parameters at the same time. The wavelength is determined by time-of-flight and the scattering angle with a position sensitive detector. This experimental arrangement improves the intensity at the sample significantly so that the reflected intensity can be measured continuously and binned down to 1 min resolution.

Annealing was performed in a specially designed rapid thermal annealing setup (AO 500, MBE-Komponenten GmbH, Germany) in argon gas. Here, extremely fast heating rates are possible which allow one to reach temperatures up to 500 °C in less than 30 s. For the *in-situ* NR experiments the annealing setup was optimized for use in the neutron beam by separating the heating chamber from the control unit and equipping it with two sapphire windows for entrance and exit of the neutron beam. The temperature of the sample was recorded during the NR measurement by a RT100 thermocouple located at the sample position and controlled by a PID controller.

Grazing-Incidence X-ray Diffractometry (XRD) for structural characterization of the samples before and after annealing was carried out using a Panalytical Empyrean diffractometer (Cu_Kα_, 40 keV, 40 mA, incidence angle α  = 1°) at the Institut für Nichtmetallische Werkstoffe, TU Clausthal.

## Results and Discussion

**Figure 1:**
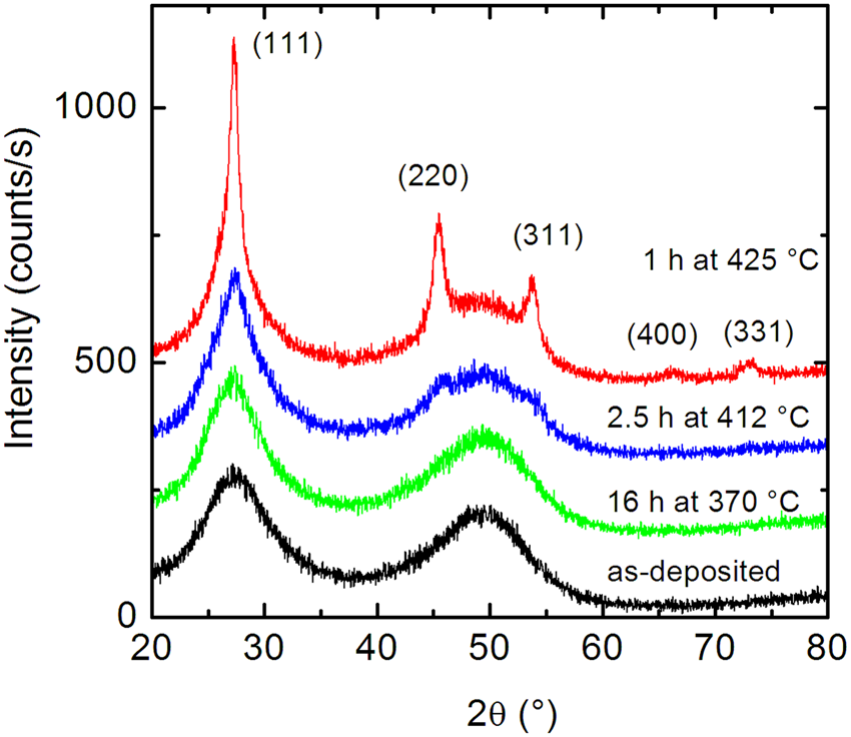
Grazing incidence X-ray diffraction patterns of [^73^Ge (14 nm)/^nat^Ge (14 nm)] × 10 multilayers at different temperatures. The patterns are shifted in intensity along the ordinate for clarity.

First, we discuss structural characterization of the samples. As can be seen from the XRD patterns in Fig. 1, the Ge multilayers are completely X-ray amorphous after deposition, as reflected in the two broad humps at 27.5 and 49.9°. Annealing up to 412 °C for 2.5 h did not change this pattern significantly. At 425 °C the main pattern is superimposed by small Bragg peaks corresponding to polycrystalline germanium with a grain size of about 10 nm as assessed by the Scherrer formula. The amount of crystalline phase can be estimated to be about 10–15% after 1 hour of annealing at 425 °C by comparing the integrated area of crystalline and amorphous parts of the diffractogram. Prolonged annealing at 425 °C for several hours leads to complete crystallization (not shown). The Bragg peaks can be indexed as (111), (220), (311), (400) and (331) in (h k l) for the cubic diamond lattice. The occurrence of all these Bragg peaks indicates that random nucleation and growth takes place. As given in^34,35,48^ crystallization of germanium takes place at about 425 °C on a time scale of 5–30 hours depending on deposition rate, film thickness and method of analysis. This indicates that the impurities found by XPS do not significantly modify the stability of the amorphous structure, e. g. by metal induced crystallization which may shift the crystallization temperature by several tens to hundreds of degrees to lower values^49^. The amount of Fe of 0.5 at.% detected here seems to be too low for a significant effect. In principle, at temperatures between 300 and 500 °C SPER can be observed if an amorphous/crystalline germanium interface exists acting as starting point for the crystallization process^50^. This is not the case as shown by Fig. 1, where random nucleation and growth is proven. This result can be traced back to the fact that the native germanium oxide layer prevents the SPER process, by omitting a direct contact. As stated in ref.^51^ the removal of the native oxide layer is critical in order to obtain successful SPER. However, it cannot be completely excluded that the detected impurities like O, Fe, C or Ar (sputter gas) may play a role of stabilization of the amorphous structure against SPER^52,53^. Consequently, the observed random nucleation and growth might be the consequence of both: the native oxide layer and/or the level of impurity concentrations.

**Figure 2:**
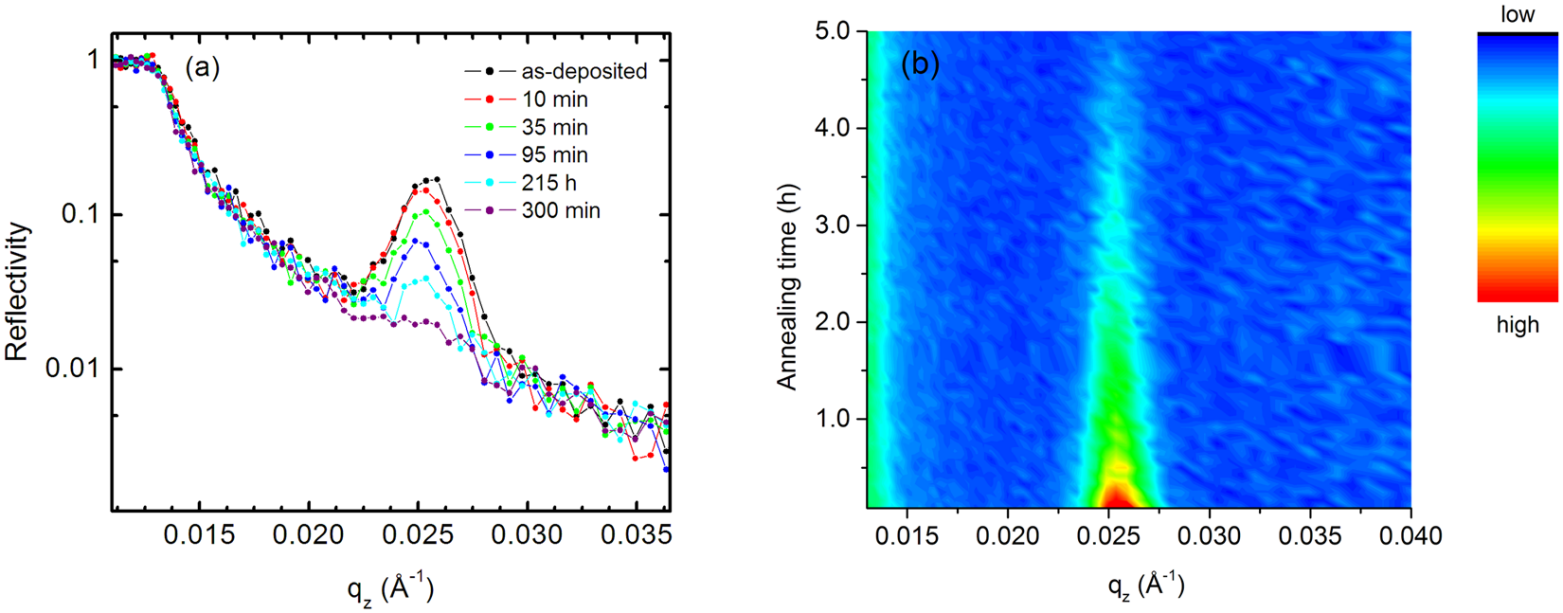
(**a**) Selected neutron reflectivity (R) patterns of ^73^Ge (14 nm)/^nat^Ge (14 nm)] × 10 multilayers for various time steps during *in-situ* annealing at 400 °C. (**b**) Contour plot of the quantity R q_z_^4^ as a function of annealing time and wave vector q_z_ (red: high relative intensity, green: middle relative intensity and blue: low relative intensity). For illustration purposes, five reflectivity patterns were averaged.

**Figure 3:**
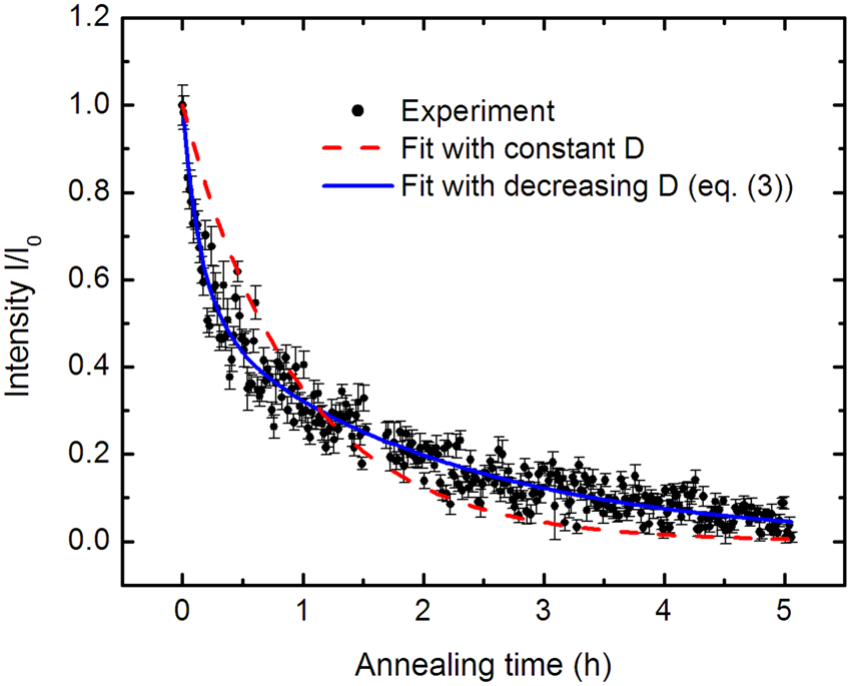
Integrated and normalized intensity of the Bragg peak, I/I_0_, as a function of annealing time at 400 °C (dots). Each data point corresponds to a subsequent annealing time of 1 min. Also given are fitting curves according to equation (1) for constant diffusivities (red dashed line) and for time-dependent diffusivities according to equation (3) (blue line).

Figure 2(a) shows the neutron reflectivity pattern of a multilayer sample in the as-deposited state. The edge of total reflection at 0.013 Å^−1^ and a Bragg peak caused by the isotope modulation at about 0.026 Å^−1^ are visible. During annealing the Bragg peak decreases continuously due to isotope interdiffusion between each of the isotope enriched single layers. The reflected intensity (reflectivity pattern) was measured continuously during isothermal annealing. The results for a temperature of 400 °C are shown in Fig. 2(a) for some selected reflectivity patterns. Overall almost 300 patterns (each accumulated for one minute) were recorded. In Fig. 2(b) a contour plot is given, illustrating the continuous decrease of the Bragg peak until complete vanishing. In Fig. 3 the relative Bragg peak intensity I/I_0_ is plotted as a function of annealing time, where I_0_ is the intensity in the as-deposited state. The quantity *I* was determined by fitting a Bragg peak with a Gaussian function after subtracting the background. In a first attempt, the diffusivity at 400 °C is obtained by fitting the data of Fig. 3 using the following equation^54^

1 $$I/{I}_{0}={\rm{e}}{\rm{x}}{\rm{p}}(-\frac{8{\pi }^{2}t}{{r}^{2}}{D}_{av}(t))$$

where, *r* = 28 nm is the ^73^Ge/^nat^Ge bilayer thickness and D_av_ is a time averaged diffusivity as given by

2 $${{\rm{D}}}_{{\rm{a}}{\rm{v}}}({\rm{t}})=\frac{1}{{\rm{t}}}{\int }_{{{\rm{t}}}_{0}}^{{\rm{t}}}{\rm{D}}({\rm{t}}^{\prime} )\,{\rm{d}}{\rm{t}}^{\prime} $$

where D(t′) is the instantaneous diffusivity. In case of a time-independent (constant) diffusivity, we get D_av_ = D. The fitting curve for a constant diffusivity is shown also in Fig. 3 as a red dashed line. As visible, the main decrease of the intensity is well reproduced. However, the fit outmatches the experimental data at low times while the reverse is valid for high times. A constant self-diffusivity of (2.41 ± 0.06) × 10^−21^ m^2^/s is derived. A better fit of the experimental data by equation (1) can be obtained (blue line in Fig. 3) if a time-dependent diffusivity is assumed. Then the diffusivity in equation (1) is given by^54^

3 $${{\rm{D}}}_{{\rm{av}}}={{\rm{D}}}_{{\rm{e}}}+({{\rm{D}}}_{{\rm{i}}}-{{\rm{D}}}_{{\rm{e}}}){\rm{\tau }}/{\rm{t}}({\rm{1}}-\exp (\,-\,{\rm{t}}/{\rm{\tau }}))$$

with an initial diffusivity of D_i_ = (8.25 ± 0.55) × 10^−21^ m^2^/s, a final diffusivity for long annealing times of D_e_ = (1.11 ± 0.04) × 10^−21^ m^2^/s and a relaxation time of τ = (0.21 ± 0.02) h. Note the low relative error limit of 4% for D_e_, resulting from the least-squares fit. For classical *ex-situ* diffusion experiments with modern methods (NR, SIMS) this quantity is significantly higher (∼15–40%)^4,16^. The bilayer thickness r in equation (1) can be determined very exactly from the location of the Bragg peak in q_z_ and the estimated error of less than 0.5% is neglected here. This low error limits in D will also lead to a reduced error in the activation energy of diffusion (see below). The time dependence of the instantaneous D is described by a first order reaction as

4 $${\rm{D}}={{\rm{D}}}_{{\rm{e}}}+({{\rm{D}}}_{{\rm{i}}}-{{\rm{D}}}_{{\rm{e}}})\,\exp (\,-\,{\rm{t}}/\tau )$$

It can be transformed into eq. (3) by inserting eq. (4) into eq. (2). A significantly improved fit of the experimental data assuming a time-dependent diffusivity was found for all temperatures under investigation in the temperature range between 370 and 412 °C.

The time-dependence of the self-diffusivities found by analysis of the experimental data can be explained with the fact that a defect annihilation process is taking place during isothermal annealing^29,55^. In general, during self-diffusion in solids a point defect-atom exchange takes place while D = f_D_ c_D_ D_D_, assuming a single dominating defect governing diffusion. Here, D is the self-diffusivity of the Ge atoms, c_D_ is the atomic fraction of the (unknown) diffusion defects, D_D_ is the diffusivity of these defects and f_D_ is the diffusion correlation factor^56^. After deposition of the multilayer and during the initial stage of annealing, a large number of non-equilibrium defects is often present in addition to the thermal equilibrium values. These defects result from a non-ideal arrangement of atoms during sputter deposition and also due to the intrinsic amorphous structure. In that case diffusion is governed by these excess point defects and the diffusivity is termed as D_i_. With increasing annealing time the number of point defects is reduced due to structural relaxation and defect annihilation at sinks^38^. This has the consequence that a decrease of diffusivities is observed. This annihilation process occurs on a time scale of the relaxation time τ. For times much larger than τ, thermal equilibrium diffusion takes place intrinsic to the metastable amorphous structure, characterized by D_e_.

An alternative explanation might be that a mass densification process of the amorphous structure during annealing may lead to a decrease of diffusivities as a function of time. NR reflectivity patterns give very exact information on the modification of the layer thickness and consequently of the density of the film under investigation. Significant densification of amorphous germanium (layer thickness decrease) will lead to a shift of the Bragg peak position in Fig. 2(a) to higher values. This is not observed. The overall thickness of the Ge multilayer as determined from the Bragg peak position varies non-systematically (statistically) during annealing by a maximum value of about 0.5%. This is not supporting the assumption of a large densification of the amorphous structure. However, this effect can definitely contribute to the decrease of diffusivities observed.

In this context, a further advantage of *in-situ* experiments over *ex-situ* experiments becomes clear. Time-dependent diffusivities with very short relaxation times of several minutes as found here cannot be identified properly by an *ex-situ* experiment. In such cases, often the effect of relaxation is averaged out and effective diffusivities are determined. Only the quasi-continuous recording of several hundred of reflectivity patterns and hence Bragg peak intensities as a function of time allows to exactly detect the time-dependence of diffusivities on the given time scale. Note also that *in-situ* experiments lead to a substantial reduction of experimental time. In principle, several hundred reflectivity patterns are recorded, each corresponding to an instantaneous diffusivity in a single experiment lasting some hours. This is not possible by *ex-situ* experiments where each diffusivity has to be measured separately. The consequences are a significant reduction of error limits and a proper identification of time-dependent diffusivities as mentioned.

Concerning literature data, Radek *et al*.^30^ investigated the interfacial broadening of amorphous Ge/^70^Ge layers prepared by self ion-implantation during SPER. An upper limit of 0.5 nm was deduced for the broadening of the multilayer interface after annealing at 400 °C for 569 s in the still amorphous part of the sample (see Fig. 2 of ref.^30^). This value can be compared to our results in Fig. 3. Using equation (1) a diffusion length of 2 (*D*_*av*_*t*)^1/2^ = 4.0 nm is found for 569 s of annealing, describing the difference between the initial and annealed state due to the decrease in neutron intensity. This is higher than the maximum value of 0.5 nm as derived by Radek *et al*.^30^. We attribute these differences to the fact that two different types of amorphous structures are prepared by different methods: Our sample by ion-beam sputter deposition between room temperature and (maximum) 80 °C and the sample of Radek *et al*. by Ge self-implantation into MBE germanium at 77 K. As indicated by our results, a substantial number of non-equilibrium point defects are likely present in our samples leading to relaxation and diffusion on the length scale of 4 nm for 569 s at 400 °C. In contrast, as stated in ref.^30^ transient local relaxation in the amorphous phase takes place without long-range diffusion (in the time domain investigated).

**Figure 4:**
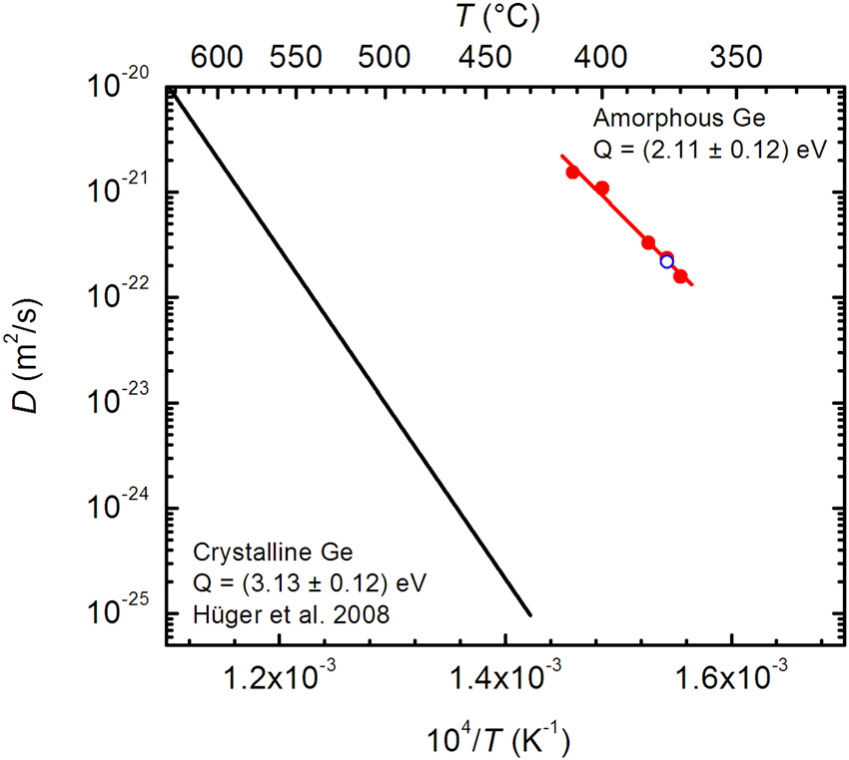
Self-diffusion coefficients determined for amorphous germanium (this work: symbols) in comparison to data reported for crystalline germanium by Hüger *et al*.^17^ (black solid line). Error limits correspond to the diameter of the dots or less. The straight red line represents a fit of the Arrhenius law (equation (5)) to the data which yields an activation energy of Q = (2.11 ± 0.12) eV. Also shown is the diffusivity for a sample pre-annealed at 425 °C for 5 min (blue open circle).

The diffusivities in metastable equilibrium D = D_e_ obtained at various temperatures are plotted in Fig. 4 as a function of inverse temperature. The diffusivities obey the Arrhenius law

5 $$D={D}_{0}\,\exp (-\frac{Q}{{k}_{{\rm{B}}}T})$$

with the activation energy of Q = (2.11 ± 0.12) eV and a pre-exponential factor of D_0_ = 6.2 × 10^−6^ m^2^/s (error: ln D_0_/m^2^s^−1^ = 2.3), while k_B_ is the Boltzmann constant. An analysis of the initial diffusivities, D_i,_ will be presented elsewhere. Note also the low relative error of the activation energy of 5% for the relatively low number of five diffusivities measured in a narrow temperature range of only 37 K. Here, the basis is laid to measure e. g. curved Arrhenius lines (if existent in some material) if temperature range and number of diffusivities is enhanced.

Also shown in Fig. 4 is a diffusivity measured at 375 °C on a multilayer that was pre-annealed at 425 °C for 5 min beforehand. This pre-annealing step leads to a decrease of the Bragg peak intensity to about 50% of the initial value. The subsequent quasi-continuous measurement at 375 °C is starting from this value. There, the experimental intensities can easily be fitted by equation (1) with a constant diffusivity in very good agreement to the Arrhenius straight line. This clearly shows that pre-annealing at higher temperatures eliminates structural relaxation effects.

A comparison of the diffusion parameters found here to those of single crystalline germanium^17^ reveals that the diffusivities in amorphous germanium are five orders of magnitude higher than in single crystals if compared at 400 °C. This large difference is mainly due to the lower activation energy of 2.11 eV in amorphous germanium compared to crystalline germanium with 3.13 eV^17^, pointing to different diffusion mechanisms in crystalline and amorphous modifications. The pre-exponential factor in amorphous germanium of 6.2 × 10^−6^ m^2^/s is relatively low and a rather conventional entropy of diffusion of *ΔS* ≈ 2 k_B_ can be assessed, using Δ*S* ≈ *ln* (*D*_0_/*a*^2^*v*) and a = 2.45 Å as the Ge-Ge atomic distance and *ν* ≈ 7.7 × 10^12^ s^−1^ as the Debye frequency^57^.

Concerning a possible diffusion mechanism, only very general statements can be given here. In general, in crystalline solids the activation energy of diffusion is the sum of a migration and a defect formation part. If this picture is adapted to amorphous germanium, these quantities are essentially unknown. For crystalline germanium, the migration energy of diffusion for the dominating vacancy defect is assumed to be below 1 eV^58,59^. For the amorphous modification the migration energy of a vacancy-like defect (if present) should be lower due to the more open structure. This implicates that the activation energy of diffusion measured here as high as 2.11 eV should be also composed of a migration and a defect formation part, if a vacancy is stable and the dominating defect. Similar as suggested for amorphous silicon^60^, diffusion may also be governed by atomic rearrangement processes via bond break or bond switch. There, atomic migration energies are also expected to be as low as 1 eV^60^. Also dangling bonds may play a role^61^. An interesting observation is that the activation energy of SPER^50^, occurring at a crystalline–amorphous interface (without native oxide layer in-between), of 2.15 eV^62^ equals our activation energy of diffusion. Since SPER is assumed to take place by local bond rearrangements a similar mechanism might be acting during self-diffusion. Such a suggestion was already made to explain Si self-diffusion in Si ion-implanted and amorphized single crystalline silicon on silicon-on-insulator wafers^4^. For SPER in amorphous germanium a bonds-switching mechanism is proposed in ref.^30^. Other possible mechanisms are discussed in ref.^63^.

In conclusion, we presented *in-situ* self-diffusion experiments in solids based on Focussing Neutron Reflectometry. Amorphous germanium was used as a model system. Diffusivities were measured quasi-continuously during isothermal annealing at different temperatures. This new approach has the advantage of a significant reduction of error limits of diffusivities while at the same time a substantial reduction of the necessary experimental time is achieved. The method also allows identification and continuous measurement of time-dependent diffusivities, which is not possible during *ex-situ* studies. The self-diffusivities in amorphous germanium follow the Arrhenius law between 375 and 412 °C with an activation energy of Q = (2.11 ± 0.12) eV with absolute values five orders of magnitude higher than in germanium single crystals at 400 °C.
